# Implicit Solvent with Explicit Ions Generalized Born Model in Molecular Dynamics: Application to DNA

**DOI:** 10.1021/acs.jctc.4c00833

**Published:** 2024-09-16

**Authors:** Egor S. Kolesnikov, Yeyue Xiong, Alexey V. Onufriev

**Affiliations:** † Department of Physics, Virginia Tech, Blacksburg, Virginia 24061, United States; ‡ Department of Biomedical Engineering and Mechanics, Virginia Tech, Blacksburg, Virginia 24061, United States; § Departments of Computer Science and Physics, Center for Soft Matter and Biological Physics, Virginia Tech, Blacksburg, Virginia 24061, United States

## Abstract

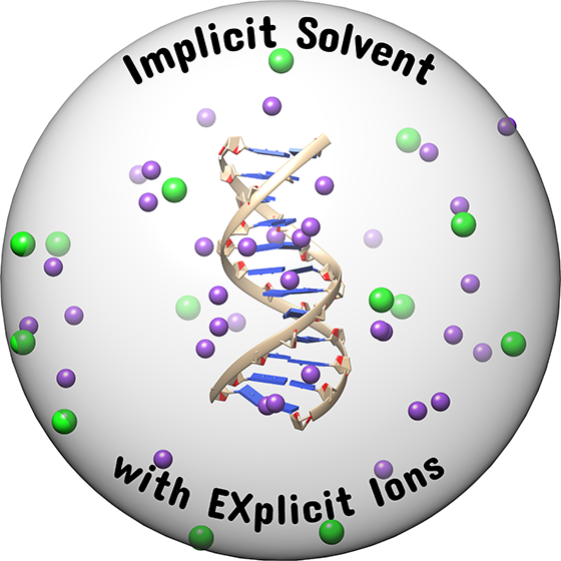

The ion atmosphere surrounding highly charged biomolecules, such as nucleic acids, is crucial for their dynamics, structure, and interactions. Here, we develop an approach for the explicit treatment of ions within an implicit solvent framework suitable for atomistic simulations of biomolecules. The proposed implicit solvent/explicit ions model, GBION, is based on a modified generalized Born (GB) model; it includes separate, modified GB terms for solute–ion and ion–ion interactions. The model is implemented in the AMBER package (version 24), and its performance is thoroughly investigated in atomistic molecular dynamics (MD) simulations of double-stranded DNA on a microsecond time scale. The aggregate characteristics of monovalent (Na^+^ and K^+^) and trivalent (Cobalt Hexammine, CoHex^3+^) counterion distributions around double-stranded DNA predicted by the model are in reasonable agreement with the experiment (where available), all-atom explicit water MD simulations, and the expectation from the Manning condensation theory. The radial distributions of monovalent cations around DNA are reasonably close to the ones obtained using the explicit water model: expressed in units of energy, the maximum deviations of local ion concentrations from the explicit solvent reference are within 1 *k*_B_*T*, comparable to the corresponding deviations expected between different established explicit water models. The proposed GBION model is able to simulate DNA fragments in a large volume of solvent with explicit ions with little additional computational overhead compared with the fully implicit GB treatment of ions. Ions simulated using the developed model explore conformational space at least 2 orders of magnitude faster than in the explicit solvent. These advantages allowed us to observe and explore an unexpected “stacking” mode of DNA condensation in the presence of trivalent counterions (CoHex^3+^) that was revealed by recent experiments.

## 1 Introduction

Ions are essential for cellular function in plants, animals, and humans. It is not surprising that even a slight imbalance of the ionic environment may disrupt cellular operations, leading to severe consequences for the organism. The key role of metal ions in various biological processes, in particular their interactions with nucleic acids, is well-documented.^1−5^ These interactions are critical in determining the mechanical properties of nucleic acids; for instance, Mg^2+^ is essential for the structural stability of RNA molecules.^6−8^ Multivalent counterions surrounding DNA and RNA molecules play critical roles in important phenomena, such as counterion-induced nucleic acid condensation,^9,10^ which is essential for chromatin compaction,^11,12^ affecting gene expression. Understanding the mechanisms behind counterion-induced condensation is important for the development of mRNA vaccines^13,14^ and gene therapies, where targeted delivery of nucleic acid payloads is challenging.^15−18^ Ion–protein interactions affect protein stability^19,20^ and regulate the activity of transcription factors.^21,22^ Ions also modulate the structure of ion-sensing proteins,^23−25^ which play roles in various physiological processes, from inflammation and smooth muscle contraction to short-term and long-term memory and immune response. Specific and nonspecific explicit ion binding is important for the structure of monoclonal antibodies.^26^ Thus, the accurate treatment of ions is just as important as the accurate treatment of water in biomolecular simulations.

**Figure 1:**
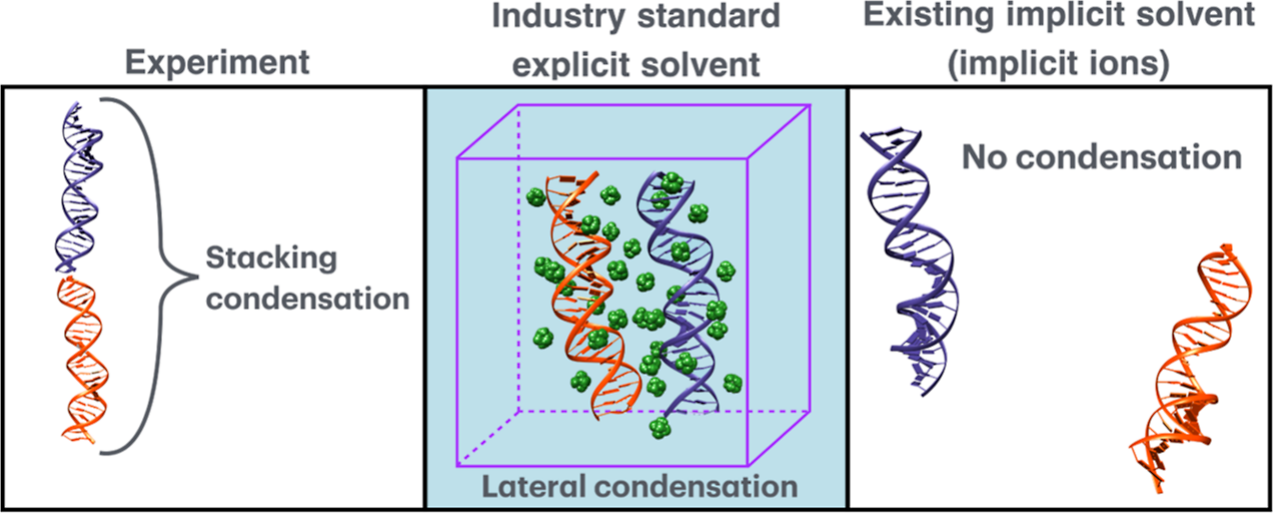
Highly approximate treatment of ions by mainstream implicit solvation models, such as the GB (right) or speed/size limitations of the traditional explicit solvent (middle), can lead to qualitatively wrong outcomes of atomistic simulations compared to experiment (left). In this example, an unusual stacking mode of DNA condensation is shown experimentally^33^ to occur for certain DNA sequences (left panel). The implicit solvation methodology that treats the ion atmosphere at a mean-field level cannot describe counterion-induced attraction and condensation of nucleic acids in principle (right panel). Only the well-known lateral DNA condensation mode is likely to be found by a standard explicit solvent simulation (middle panel) due to the high computational costs associated with the significantly larger system size and simulation times needed to reach the stacking condensation mode.

Atomistic molecular dynamics (MD) simulations are one of the most widely used theoretical tools in biomedical research,^27−30^ which requires accurate and, at the same time, computationally facile representations of the key elements of aqueous solvation–ions and water. Among existing classical models of aqueous solvation, the most accurate ones treat individual solvent molecules and biologically relevant ions explicitly, on par with the target biomolecule. However, this level of detail often entails a significant computational cost, making some simulations difficult or impractical. For instance, close to microsecond simulation times are necessary to properly average various parameters in nucleic acid systems with multivalent ions.^31,32^ A system that contains multiple DNA molecules that can form unusual states of counterion-induced condensation, Figure 1 (left), may require a very large solvent box to model properly; a typical “parsimonious” setup where the box is just big enough for the expected but wrong state, Figure 1 (middle), will miss the right one in a typical simulation. In general, an unanticipated conformational transition leading to a significant increase of the size of the “structural unit” would likely be missed in a parsimonious solvent box designed to simulate the original, smaller system.

Apart from the general issue of computational expense and the relatively slow speed of conformational sampling in the explicit solvent,^34^ the traditional explicit solvent approach has several other, less obvious, but highly significant limitations. Simulating physiologically relevant (intracellular) conditions of small concentrations of many multivalent ions,^35,36^ e.g., 1 mM of free Mg^2+^ or 0.1 mM of Ca^2+^, in the bulk solvent around a macromolecule is challenging to impossible in a reasonably sized box of explicit water as the number of explicit water molecules required to produce the necessary dilution of even a few tens of unbound ions in the bulk becomes enormous.

The implicit solvation model, an efficient alternative to explicit solvent methods, accelerates conformational sampling in simulations of biomolecules.^34^ It represents the solvent as a continuum, capturing water’s dielectric and nonpolar properties,^37−47^ and is grounded in solid physical principles.^48^ Currently, the generalized Born (GB) model,^42,49−79^ a variant of implicit solvation, is the de facto “engine” of molecular simulations in the implicit solvent,^a^ where it enhances computational and sampling efficiency in MD.^86−118^

The GB is a “trail blazer” method, which can justify the additional approximations to reality made^119^ by this model relative to the traditional explicit solvation. For example, it was the GB model that enabled early simulations^87−96^ of “de novo” protein folding, long before the advent of highly specialized supercomputers made it possible in the explicit solvent.^120^ Atomistic folding simulations using the GB model are now routine on GPUs,^121^ accessible to most computational laboratories. The GB-based MD simulations are particularly well suited for modeling of large-scale motions in biomolecules,^97,98^ including the nucleosome and its DNA;^99^ for the latter, speed-ups of conformational sampling up to 2 orders of magnitude can be expected.^34^ Recent GPU implementations^122^ have significantly boosted the GB model’s performance, enabling faster simulations^34,123^ of large^124^ structures previously challenging for explicit solvents.

**Figure 2:**
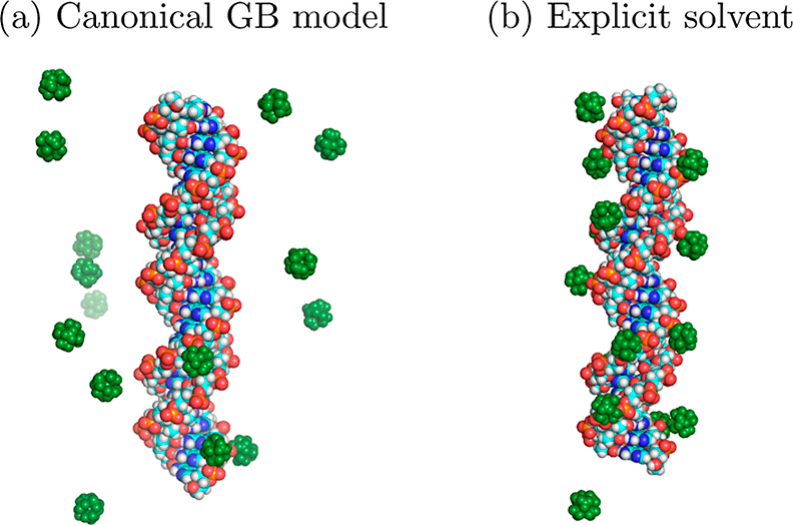
An MD simulation based on the canonical GB model generates a qualitatively wrong distribution of multivalent ions around DNA (a), in which explicit trivalent ions (CoHex^3+^, green) fail to condense onto the double-stranded DNA. The expected counterion condensation is reproduced by a fully explicit solvent (b). The distributions are adapted from ref (127). The qualitative inadequacy of CoHex^3+^ distribution seen in (a) stems from a fundamental limitation of the canonical GB model when applied to disconnected, i.e., water-separated, topologies,^127^ such as ions around a molecule.

Yet, the canonical GB framework,^53^ foundational to various GB flavors^125^ (models) available in major simulation packages, has a notable limitation: it is designed to approximate the effects of monovalent ions alone, representing them as a diffuse, mean-field continuum (Debye screening), neglecting ion correlation effects, as well as differences between ions such as Na^+^ versus K^+^,^154^ or specific ion binding. Multivalent ions remain out of reach. Electrostatic potential distributions around biomolecules, obtained with explicit ions,^31,126,127^ often differ significantly from those produced using more approximate ion models such as the GB, which treat ions as a diffuse continuum. The fundamental limitations have their consequences for practical simulations: for example, the canonical GB model is not suitable for simulations of the DNA condensation process, see Figure 1. Straightforward attempts to use explicit ions directly within the canonical GB lead to qualitatively wrong results, see Figure 2. Collectively, these limitations make the canonical GB unsuitable for high-accuracy simulations of many biologically relevant systems and processes—all where detailed ion interactions are crucial. The very approximate treatment of ions by the current GB model was likely adequate for a number of problems at much shorter time-scales of yesterday; we argue that it is no longer the case today.

Recognizing this gap, here we introduce the Implicit Solvent with EXplicit Ions approach and its practical implementation GBION, which enhances the capabilities of atomistic simulations by combining explicit ion treatment with the benefits of implicit water solvation. This work lays out the foundation of the new model; as a concrete example of its potential application here, we focus on the important case of DNA.

## 2 Theory

### 2.1 Canonical GB Model: Brief Introduction

The hydration free energy plays a pivotal role in determining the interaction potential between solutes in environments characterized by water. This energy, denoted as Δ*G*_solv_, can be decomposed into two main contributions: electrostatic, Δ*G*_el_ , and nonpolar, Δ*G*_non-polar_, contributions^128^
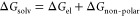
1which are typically estimated independently. A widely used, computationally efficient approximation to calculating the nonpolar term (Δ*G*_non-polar_) is based on the assumption that Δ*G*_non-polar_ is proportional to the solvent-accessible surface area (SASA).

The GB model provides an approximate way, relative to the exact solution of the Poisson equation, to calculate the electrostatic part, Δ*G*_el_, of the solvation free energy of any singly connected solute or tightly bound complex.^119^ The canonical GB approximation is based on the equation originally proposed by Still et al.^53^
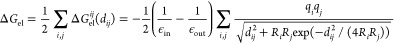
2where ϵ_in_ and ϵ_out_ are the dielectric constants of the solute and the solvent, respectively, *d*_*ij*_ is the distance between solute atoms *i* and *j*, and *q*_*i*_ and *q*_*j*_ are the atomic charges. The main parameters of the equation are the effective Born radii *R*_*i*_, with *R*_*i*_^–1^ characterizing the average degree of solvent exposure of atom *i*. Generally, *R*_*i*_ is larger than the atom’s intrinsic radius ρ_*i*_, the two becoming equal for a single isolated ion or atom. Multiple practical algorithms^125^ exist for calculating *R*_*i*_ from the molecular geometry defined by a set of its {ρ_*i*_} and interatomic distances; fast analytical flavors of the GB are most often used in MD simulations.

### 2.2 Proposed GBION Model

The canonical GB model^125^ exhibits remarkable accuracy when applied to single, topologically connected molecular structures. This accuracy arises from the fact that these structures are topologically equivalent to a sphere, where the GB model becomes essentially exact in the limit of the infinite solute dielectric.^129−131^ Conversely, it is not surprising that the model’s accuracy deteriorates for topologies distinctly different from a sphere: “disconnected” topologies, such as solvent-separated molecules. Understanding and simulating these “disconnected” topologies are critical for elucidating various biologically relevant processes, including ligand binding and intricate interactions of DNA with the surrounding ions.

A general approach, suitable for Monte Carlo simulations of disconnected solutes, was proposed earlier by Tolokh at al.^127^ However, that approach implies a discontinuous, and therefore nondifferentiable, solvation energy function. Consequently, forces cannot be calculated, and the approach cannot be implemented “as is” in MD simulations. A version with a differentiable energy Δ*G*_el_^*ij*^ is presented here.

**Figure 3:**
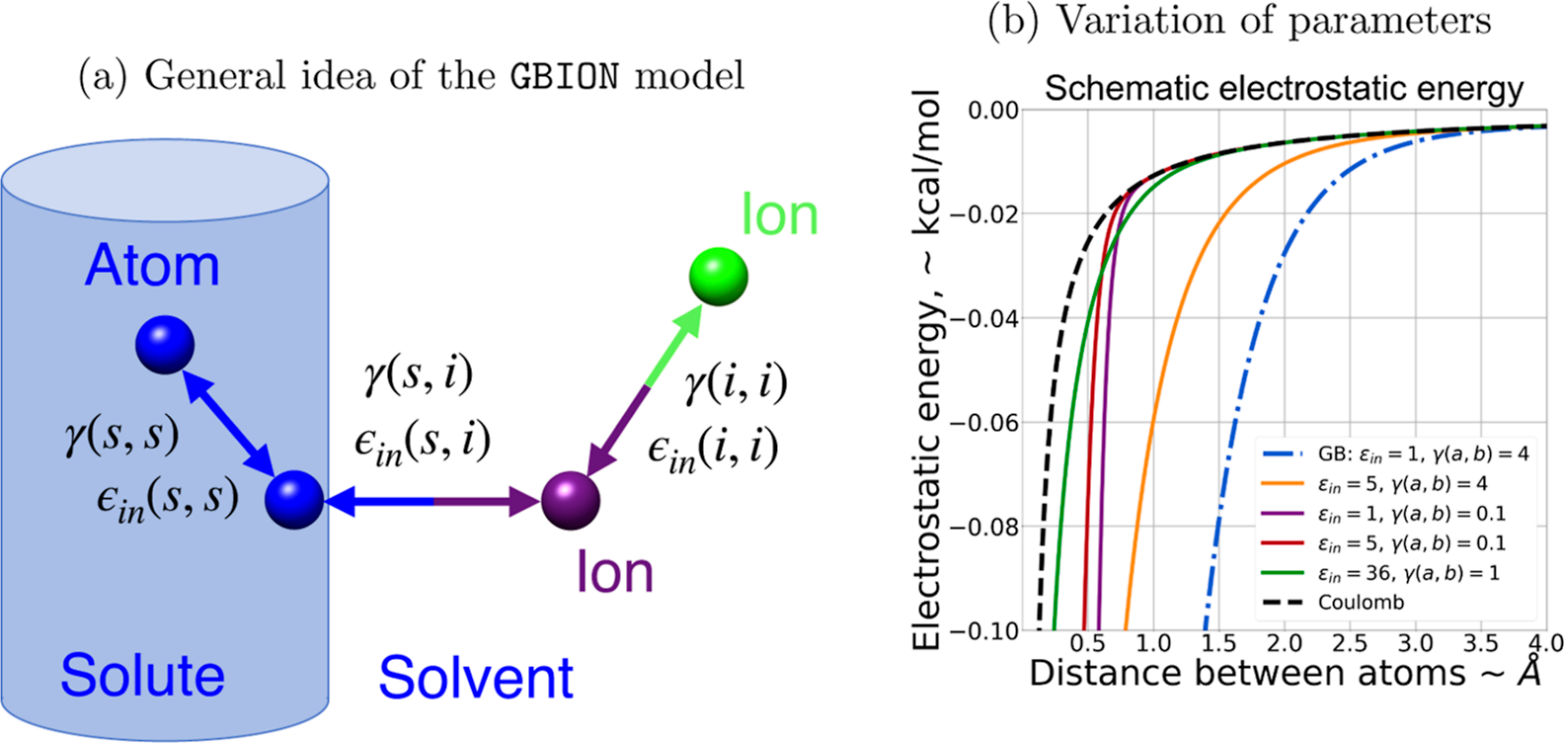
(a) Adaptation of the general implicit solvent/explicit ions framework for MD simulations. Each pair (a,b) of interacting atom types has its own parameter set describing their interactions, eq 3. (b) Dependence of the total electrostatic energy on the distance *d*_*ij*_ between two charged atoms for different values of γ(*a*,*b*) and ϵ_in_(*a*,*b*). Decreasing γ(*a*,*b*) makes the interacting potential closer to Coulomb’s law with a stronger effect for larger values of *d*_*ij*_. The smaller the γ(*a*,*b*), the closer *E*_*ij*_(*d*_*ij*_) is to the pure Coulomb electrostatics (black dashed line). Increasing ϵ_in_(*a*,*b*) from 1 to ϵ_out_ makes *E*_*ij*_(*d*_*ij*_) deviate further from the canonical GB and closer to Coulomb’s law for all *d*_*ij*_. In all of our calculations, we use the default AMBER value of ϵ_out_ = 78.5. An example functional dependence for one of the optimized parameter pairs [ϵ_in_(a,b) = 36 and γ(a,b) = 4] is also shown (green solid line).

We begin here by identifying a new charge-pairwise Δ*G*_el_^*ij*^ suitable for multiconnected geometries (disconnected solutes) to replace the Δ*G*_el_^*ij*^ of the canonical GB, eq 2. The key requirements are that the resulting functional form is differentiable and “simple” enough to make adaption for MD possible. In contrast to the canonical Δ*G*_el_^*ij*^, the new function depends on the topology of the interacting atoms (i.e., ion–ion, ion–solute, or solute–solute), see Figure 3. The total interaction electrostatic energy for two charges with the proposed Δ*G*_el_^*ij*^ takes the form
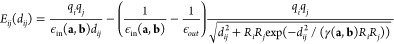
3where (**a,b**) denotes the type (topology) of the pair of interacting atoms, e.g., **a** = ion and **b** = solute. This equation captures the general nature of charge–charge interactions mediated by the solute or solvent, introducing γ(a,b) and ϵ_in_(a,b) coefficients for tuning the interaction energy. The parameter γ(a,b) controls how the function approximates the interaction between solvent-separated charges (see Figure 3); it was shown previously^127^ that, unlike the canonical GB (γ(*a*,*b*) = 4 in eq 3), pure Coulomb interaction works reasonably well for solvent-separated charges, including ions^b^. In eq 3, the pure Coulomb regime is approximated by setting γ(a,b) ≪ 1; note that  when γ(a,b) → 0. Additional flexibility to fine-tune charge–charge interactions is afforded by the coefficient ϵ_in_(a,b), which scales the part of the electrostatic energy (see Figure 3). Increasing ϵ_in_(a,b) from 1 to ϵ_out_ makes *E*_*ij*_(*d*_*ij*_) further from canonical GB and closer to Coulomb’s law for all *d*_*ij*_. The introduction of specific coefficients γ(a,b) and ϵ_in_(a,b) for interactions between solute atoms and ions (both anions and cations) enables the model to fairly accurately mimic the localized effects of ions on solvation energetics. For solute–solute interactions, γ(a,b) = 4 and ϵ_in_(a,b) = 1, meaning that the canonical GB is used.

### 2.3 Setting the Salt Concentration within the Model

**Figure 4:**
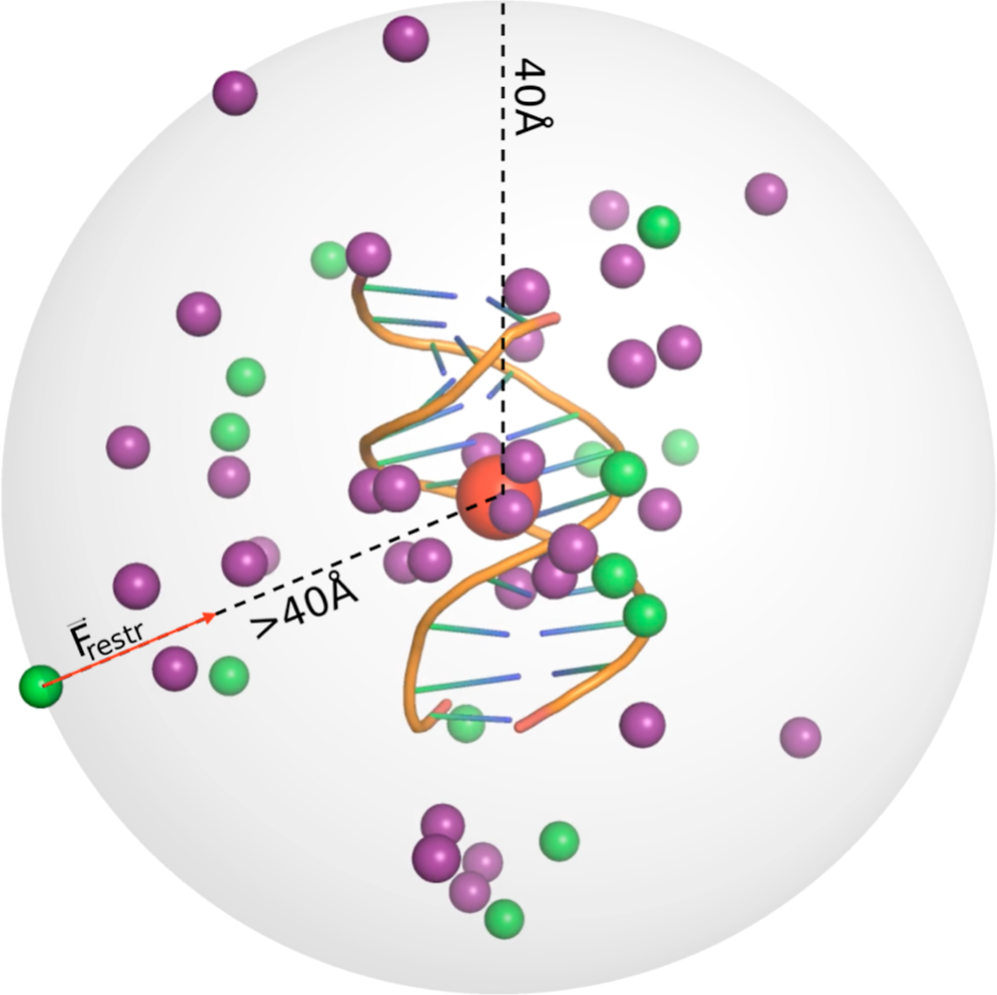
Setting the simulation volume and proper ion concentration in GBION. A restraining force *F*_restr_ (red arrow) prevents the ions (purple and green) from drifting beyond a user-specified distance (40 Å in this example) from the anchor point (center of the red sphere). Shown is an example of the force restraining the ions to the center of mass of all of the phosphorus atoms of the DNA fragment. The snapshot is taken from a simulation using GBION, see the movie in the Supporting Information.

Since there are no periodic boundary conditions in this implicit GB solvent model, an explicit ion in such a system would not be “imaged” back as it moves away from the solute. Consequently, over extended simulations, ions would gradually drift away from the solute; given an infinite volume and a finite number of particles, entropy always prevails. To address this issue, we have applied an NMR-type restraint between each ion and a user-selected “anchor”, which can be either a single atom near the center of the solute molecule or the center of mass of a group of atoms, see Figure 4 and the movie in Supporting Information. The restraint operates as follows: once the ion exceeds a predefined distance (parameter *r*3 in the AMBER *DISANG* file) from the anchor point, the ion begins experiencing a harmonic restraining potential (switched on by *nmropt* setting in AMBER and specified in the *DISANG* file): the force constant is chosen to be large enough so that possible excursions of the ion beyond the distance *r*3 should be negligible compared to *r*3. This approach allows one to define the volume of the simulation system as the volume of a sphere of radius *r*3, see the example of the simulation system with the restraining sphere in Figure 4.

To set the ion concentration in a simulation, we use the SLTCAP^132^ method. It utilizes the equation below to calculate the numbers of anions and cations that should be added to the system
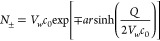
4where *N*_+_ is the number of cations to be added, *N*_–_ is the number of anions to be added, *Q* is the total charge of the molecule (solute), *V*_w_ denotes the volume of the solvent in the system in Å^3^, and *c*_0_ is the desired salt concentration in units of particles per Å^3^; to convert from the common M/L, one should multiply the M/L value by 6.02 × 10^–4^. The solvent volume *V*_w_ is defined as the volume of the enclosing sphere minus the volume of the solute. For instance, in the case of our example restraining sphere (see Figure 4), the volume , where *V*_solute_ is the volume of simulating DNA. Substituting 40 Å for *R* yields  and 
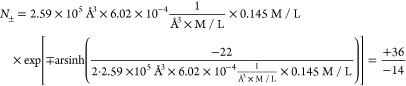
In this specific example, we add 36 cations and 14 anions to achieve the bulk salt concentration of 0.145 M in the simulation volume.

However, in practice, it is often the case that *V*_solute_ ≪ *V*_w_, and therefore one can simply neglect the solute volume and calculate the solvent volume *V*_w_ as the sphere volume. Consequently, in most cases, one can use the approximate equation below to calculate the numbers of cations and anions
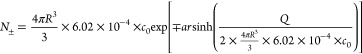
5where *R* denotes the radius of the confining sphere in Å and *c*_0_ is the desired salt concentration in M/L. If the formula returns noninteger *N*_+_ and *N*_–_, one should take the closest integer for either *N*_+_ or *N*_–_ and subtract it from the absolute value of *Q* to find *N*_–_ or *N*_+_, respectively.

## 3 Proposed Implicit Solvent/Explicit Ions GB Model Applied for Simulations of DNA

To evaluate the proposed implicit solvent/explicit ions model, we focus on its ability to reproduce ion distributions around highly charged DNA molecules in water as well as the ability of the method to maintain the proper structure of the DNA in the simulation. Specific validation involves comparing the distributions of cations around DNA with those calculated in explicit solvent simulations and examining the DNA structure’s stability during the simulation with the GBION model. The similarity of ion distributions and the preservation of DNA structure over extended periods emphasize the GBION model’s suitability for MD simulations of DNA. Additionally, we assess the computational efficiency of the GBION model relative to traditional explicit water models.

Given the propensity of DNA to condense in solutions containing ions with a charge of +3e or higher,^9,133^ the model’s ability to simulate DNA condensation processes is crucial – testing the model for the expected DNA association in the presence of CoHex^3+^ ions is another critical test. As we shall see below, our simulations reveal not only the expected lateral but also, notably, “stacking” condensation of DNA duplexes—a phenomenon only very recently observed experimentally.^33^

First, we compare distributions of sodium and potassium ions around DNA obtained using the GBION model with the corresponding distributions obtained using the explicit water model and check the stability of the DNA in these simulations. Following this, the GBION model is applied to simulate double-stranded DNA in the presence of trivalent ions (CoHex^3+^); we perform a qualitative comparison of the distribution to the published results. To check the applicability of the GBION model for the simulation of DNA condensation, we simulate a pair of DNA duplexes in the presence of CoHex^3+^ ions to observe DNA attraction. We observe lateral condensation of DNA duplexes, which agrees with experimental results.^9^ We also unexpectedly observe a “stacking” condensation mode of DNA duplexes. That condensation mode has been revealed recently^33^ (and was not known to us when we ran the simulations). Details of the simulations and comparisons are presented below.

### 3.1 Distributions of Monovalent Na^+^ and K^+^ Ions around the DNA Duplex

We use GBION to calculate monovalent Na^+^ and K^+^ ion distributions around the homopolymeric 25 base pair (bp) DNA duplex with the sequence: GCATCTGGGCTATAAAAGGGCGTCG. First, we have compared the overall characteristics of cation distributions around DNA obtained using the GBION model and explicit water models with each other and with available experimental results, see Table 1. The aggregate characteristics we compare with available references are the number of ions associated with the DNA, ion affinity for the DNA, and the degree of DNA charge neutralization. For the number of sodium ions associated with the DNA, we used the results of previous theoretical and experimental studies^134^ and our recent simulations. The degree of DNA charge neutralization is compared with the results of the explicit solvent simulations and with predictions of a well-established theory.^135^

**Table 1:** Aggregate Characteristics of Cation Distributions around DNA Obtained Using GBION Are Compared with Those Based on Explicit Water Models. Reference Values from the Experiment^134^ and Manning Condensation Theory^135^ Are Shown Where Available. The GBION Model Reproduces the Aggregate Parameters of Cation Distributions with Reasonable Accuracy.^a^

	number of cations associated with DNA		degree of DNA charge neutralization, %		cation affinity, *k*_B_*T*	
water model	Na^+^	K^+^	Na^+^	K^+^	Na^+^	K^+^
OPC	30.8 ± 0.5	30.9 ± 0.5	56 ± 4	54 ± 3	–2.28 ± 0.02	–2.22 ± 0.02
SPC/E	33.7 ± 0.5	N/A	62 ± 4	N/A	–2.45 ± 0.02	N/A
GBION	33.7 ± 0.5	34.2 ± 0.5	53 ± 2	54 ± 2	–2.07 ± 0.02	–2.09 ± 0.02
experiment	37 ± 2	N/A	N/A	N/A	N/A	N/A
manning theory	36.5	36.5	76	76	N/A	N/A

a The deviations are calculated as standard errors of the mean.

One can see that the aggregate characteristics of the cation distribution around DNA obtained using the GBION model are close to the ones obtained using the explicit water model. A previous study^136^ demonstrated that simulations using widely adopted explicit water models can approximate the number of Na^+^ ions associated with DNA to within ∼12% of the experimental value. The GBION model achieves a comparable level of agreement with the experiment, Table 1. It is important to note that differences between specific details of the simulation protocol can influence the simulation outcomes within that level of accuracy; these details include the types of restraints used (e.g., with the entire DNA restrained as in this work vs only the middle base pair kept fixed as in ref (136)) and the geometry of the simulation enclosure (e.g., a truncated octahedron box for explicit water simulations in this study vs a rectangular box in ref (136) vs a restraining sphere for GBION simulations). With this caveat in mind, one can reasonably conclude that GBION reproduces the aggregate characteristics of the cation distribution around DNA at the same level of accuracy that may be expected from the established explicit water models.

**Figure 5:**
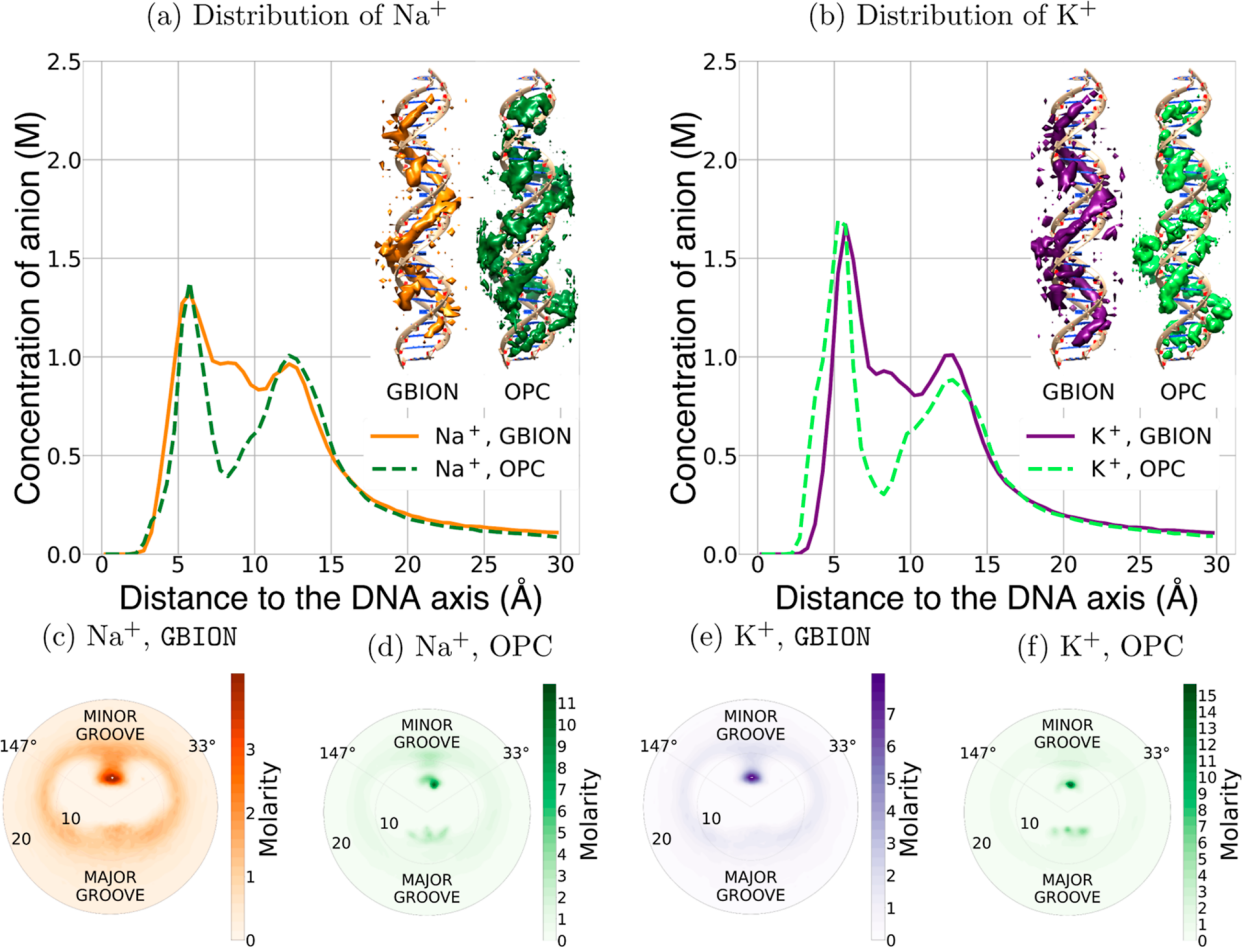
Radial distributions and ion density maps of Na^+^(a) and K^+^(b) obtained using the GBION model are compared with those obtained using the explicit solvent [OPC water^137^ with J/C (Joung/Cheatham) ion parameters^138^]. Radial-angle distributions of Na^+^ and K^+^ are presented in panels (c–f); the water model used to obtain each distribution is indicated above the panels. The distributions of sodium and potassium around DNA obtained with the GBION model reproduce those obtained using the explicit solvent with reasonable accuracy, with even the most significant difference corresponding to a variation in the ion-DNA affinity of less than *k*_B_*T*. The GBION and explicit solvent distributions in panel (a) differ the most at a distance of ∼8 Å from the DNA helical axis, the same as those in panel (b).

Below is a more detailed comparison of the distributions of sodium and potassium obtained using GBION with the ones obtained using simulations with the explicit water model OPC, see Figure 5. One can see that the ion distributions generated by GBION reproduce the explicit water MD results with a reasonable accuracy, comparable with the accuracy of the explicit water simulations themselves.^136^ The uncertainty of the explicit solvent models in reproducing the ion distributions can be estimated by comparison with the corresponding distributions obtained by using different established explicit water models. Specifically, in ref (136), the SPC/E water model was found to yield ion distributions that differ the most from those obtained via other water models tested in that work. To estimate the uncertainty of the ion distribution expected from an explicit water model, we have compared the distribution of Na^+^ ions obtained using different explicit water models (see Figure S1). The uncertainty can be estimated as the ratio of the ion concentrations at the distribution peaks. The concentration of Na^+^ ions at the first peak in the SPC/E water model is about 1.6 times higher than that obtained in the OPC water. This concentration difference translates into the difference of Na^+^-DNA interaction potentials for these two explicit water models of an order of 0.55 × *k*_B_*T*. Another representative metric of uncertainty can be the ratio between the areas integrated under the first peak of the ion distributions obtained by using different water models. In our simulations, this ratio is 1.62, consistent with the uncertainty estimate based on the peak concentration ratio estimated above. The uncertainty puts in perspective the accuracy of the proposed GBION model: the largest difference between the GBION-derived ion distribution and the one obtained using the OPC water model, Figure 5, is still less than *k*_B_*T* in energy units, which is reasonable and comparable to the difference between the ion distributions obtained in SPC/E vs OPC explicit water models.

The most noticeable difference between the distributions of ions obtained using the GBION model and explicit solvent is at a distance of ∼8 Å from the DNA helical axis. As observed from the radial-angle ion distributions shown in Figure S2, the distributions of sodium and potassium ions at approximately 8 Å from the DNA helical axis are more diffuse in GBION simulations compared to those in the explicit water simulations. The distributions reveal two binding regions, one inside and one outside the minor groove. In the explicit water simulations, there are almost no ions present between these regions. In contrast, GBION simulations show a significant presence of ions in the gap between the binding areas. It is this difference in ion distributions within the minor groove that accounts for the observed discrepancy between the radial distributions obtained using the explicit water model and the GBION model. Importantly, however, the overall number of ions bound to the DNA does not differ appreciably between GBION simulations and the explicit solvent ones. In fact, the difference is no more than 2 ions, which corresponds to ∼4% of the degree of DNA charge neutralization. This small difference is less than that between different explicit solvents^136^ in the same context. It means that the GBION model correctly captures the overall physics of ion binding to the DNA. We have also calculated the distributions of Cl^–^ around DNA in simulations of DNA in the presence of NaCl and KCl (see Figure S3). The distributions obtained in GBION are close to those in the explicit solvent and are insensitive to the cation type present in the solution.

A crucial advantage of the implicit compared to the explicit solvation is the high speed of conformational sampling that the implicit solvent model offers.^34^ We find that the proposed GBION retains this advantage. To estimate how fast the ion distribution equilibrates with GBION, we have compared the distributions averaged over different time intervals: over 10 ps, 100 ps, 1 ns, and 10 ns for GBION simulations and over 100 and 300 ns for the explicit water simulations (see Figure S4). According to our estimates, the ion atmosphere converges at least 2 orders of magnitude faster in GBION than in the explicit solvent.

### 3.2 DNA Remains Stable in GBION MD Simulations

**Figure 6:**
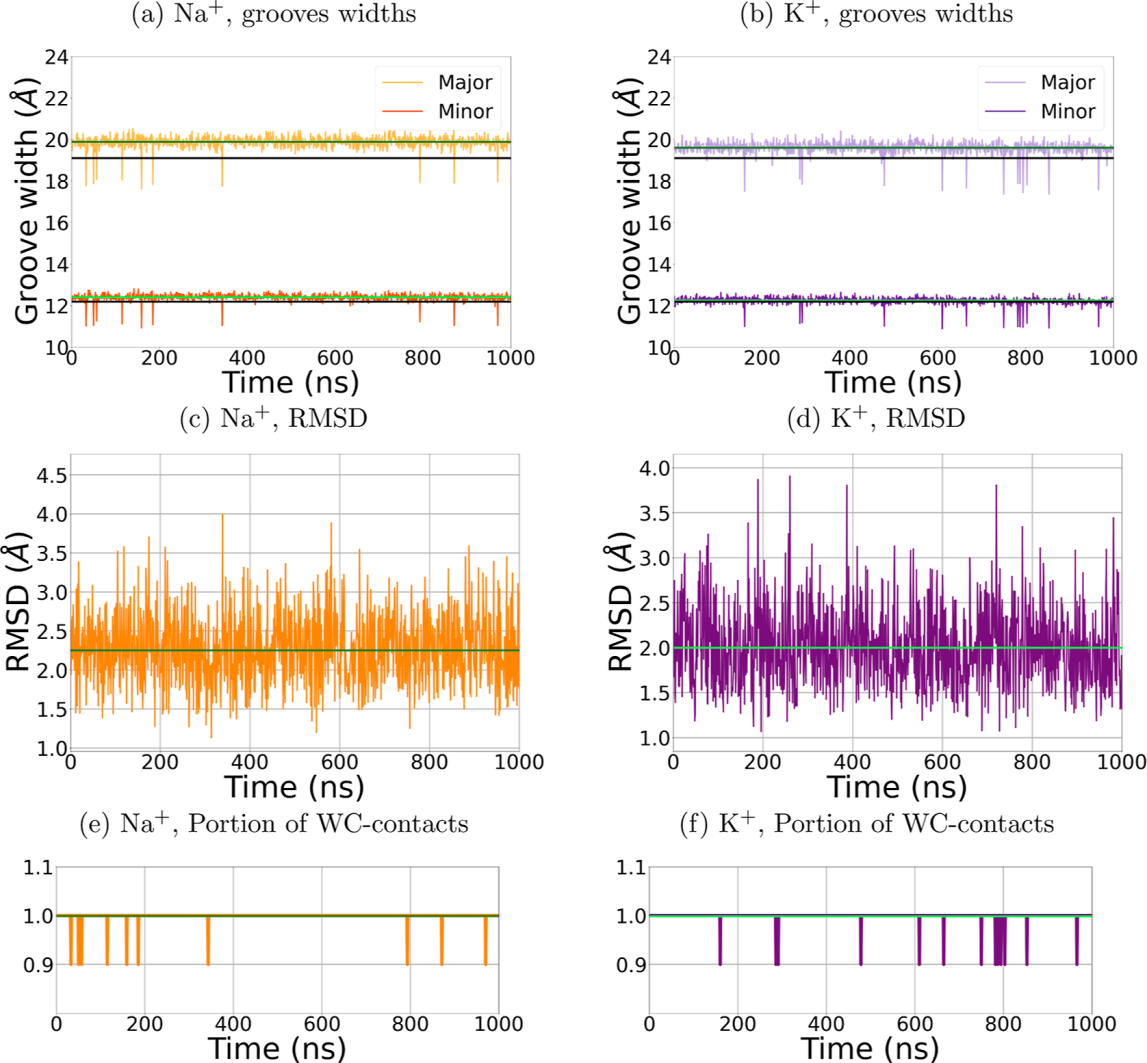
(a,b) Widths of the major (light orange and light purple) and minor (dark orange and dark purple) grooves in comparison with the experimental values^139^ (black lines) in GBION simulations with NaCl (a) and KCl (b). Average values over the simulation time are indicated by green lines. The widths of the grooves agree with the corresponding experimental values. (c,d) All-atom (excluding terminal nucleotides) rmsd to crystal structure reference in GBION simulations with NaCl (c) and KCl (d). (e,f) Fraction of stable WC-pairs (averaged over the DNA and excluding the terminal nucleotides) in GBION simulations with NaCl (e) and KCl (f).

To verify that the DNA structure remains stable in a typical MD simulation, we have simulated Dickerson–Drew dodecamer^139^ using the GBION model for 1 *μs* in the presence of NaCl and, separately, of KCl, at physiologically relevant conditions. Throughout the simulations, the ions were restrained to remain within 40 Å of the center of mass of the phosphorus atoms of the DNA using a restraining force (see an example of the restraining force in Figure 4 and a movie in the Supporting Information). We assessed the DNA stability using three key metrics: the average portion of stable Watson–Crick (WC)-pairs, rmsd from the X-ray reference, and the widths of the DNA grooves, all evaluated over the entire microsecond-long trajectory, see Figure 6.

The average WC-value was 0.99 ± 0.03 for NaCl and 0.99 ± 0.04 for KCl, indicating that the WC pairs remained stable throughout the simulation. The minor groove width in the presence of NaCl and KCl was 12.4 ± 0.2 and 12.2 ± 0.2 Å, respectively. The width of the major groove in the presence of NaCl and KCl was 19.9 ± 0.3 and 19.6 ± 0.3 Å, respectively. These widths are in agreement with those calculated from the reference structure^139^ (12.2 for the minor groove and 19.1 Å for the major groove), see Figure 6. The average rmsd from the X-ray reference structure was 2.0 ± 0.5 Å for NaCl and 2.3 ± 0.5 Å for KCl, respectively, slightly higher than the results obtained with the explicit water model (1.5 Å). The consistency of the structural parameters of the simulated DNA with the experimental references demonstrates the DNA structure’s stability during simulations with the GBION model, confirming the model’s applicability to simulating DNA duplexes in the presence of monovalent ions.

### 3.3 Distribution of Trivalent CoHex Ions around Double-Stranded DNA

To evaluate the suitability of the proposed implicit solvent/explicit ions GB method for simulating solutions with multivalent ions surrounding DNA, where ion–ion correlations and other explicit solvent effects may significantly affect the ion distributions,^140^ we applied the GBION model to investigate the interactions between trivalent CoHex^3+^ counterions and DNA duplexes. Accurately reproducing the distributions of multivalent ions around nucleic acids is crucial as subtle aspects of these distributions influence the propensity of nucleic acids to aggregate.^31,141^

**Figure 7:**
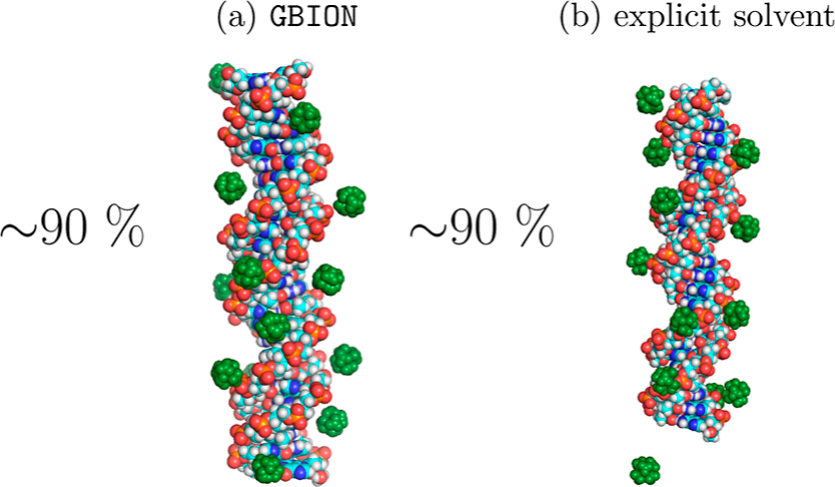
Snapshots from simulations of DNA in solution with CoHex^3+^ show the distribution of CoHex^3+^ ions (green) in simulation with the GBION model (a) and previously reported explicit solvent distribution^31,142^ (b), which agree with each other quantitatively. The degree of DNA charge neutralization obtained in the simulation carried out using the GBION model is equal to the one observed in the simulation with the explicit water model; the corresponding values are listed next to each structure.

The CoHex^3+^ ion distribution around a DNA duplex, as determined by GBION simulations, is compared with these from all-atom MD simulations utilizing the explicit water model, Figure 7.

The CoHex^3+^ distribution estimated based on the simulations carried out using the GBION model, as one can see in Figure 7, qualitatively reproduces the distribution of trivalent ions obtained using the explicit water model. The degree of DNA charge neutralization matches the one observed in simulation of DNA with CoHex^3+^ using the explicit water model^142^ (∼90%).

According to the previously developed multishell model of counterion-induced condensation of nucleic acids,^31,142^ CoHex^3+^ ions bound within the “external” ion-binding shell (12–16 Å from the helical axis) of the double helix facilitate the condensation. The probability of condensation increases with the number of ions attached to the external shell. The DNA charge neutralization is also a prerequisite for condensation. Our next step involves verifying whether DNA condensation occurs in the presence of trivalent ions, further assessing the effectiveness of GBION.

### 3.4 Counterion-Induced DNA Condensation

To assess the applicability of the GBION model for simulating the DNA condensation process, we employed the GBION model to simulate the condensation of DNA induced by CoHex^3+^ ions. The simulation began with the two DNA duplexes positioned at a distance from each other, followed by the removal of constraints to allow for the DNA attraction to lead to the condensation. The initial conformation and the observed condensed states are illustrated below.

**Figure 8:**
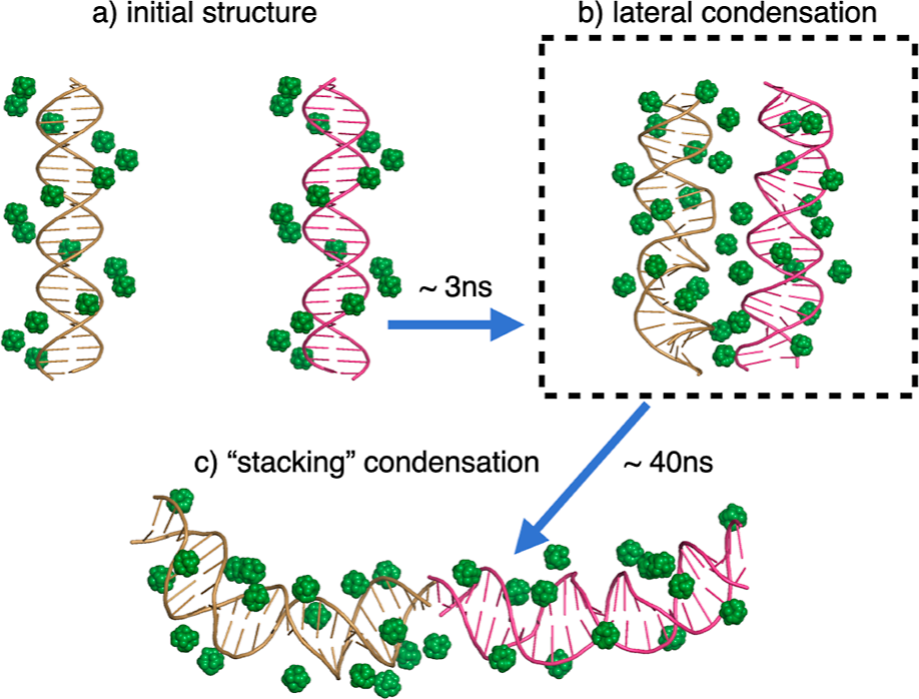
DNA condensation induced by CoHex^3+^ ions. Starting from two separated duplexes (a), a GBION MD simulation reaches the expected “lateral” condensed state (b) on a nanosecond time scale. Continuing the GBION run predicts an unexpected, novel “stacking” condensed state (c), recently validated experimentally.^33^ This state would likely not have been identified in a conventional explicit solvation simulation, which would likely use the solvent box (black dashed outline) just large enough to contain the anticipated lateral configuration of the duplexes. A movie is available in Supporting Information.

During the simulations, we identified two distinct conformational states of condensed DNA: the expected lateral condensation and a “stacking” condensed state, see Figure 8. The latter has been observed and studied in highly concentrated DNA solutions.^33^ It was also observed using SAXS.^143^ The simulation time required to observe the “stacking” condensed state in GBION is just ∼43 ns. For this simulation, we have calculated the average Watson–Crick (WC) value over both duplexes, finding it to be 0.99 ± 0.03, which indicates that the DNA remained stable throughout the condensation process induced by trivalent ions.

As we have already mentioned, sampling of the conformational space by the system in simulations carried out using the GBION model is faster than that of the explicit water simulations by 1.5–2 orders of magnitude, or more. This speed up translates, e.g., the simulation time of ∼43 ns using the GBION model, Figure 8, into ∼4 μs of effective simulation time in the explicit water. Also, to observe the “stacked” condensation mode, the simulation box for the system with the explicit water should also be large enough to accommodate two stacked DNA duplexes. A back-of-the-envelope estimate^c^ suggests that to observe the stacking condensation mode in the explicit solvent, one would need at least ∼0.5 years on the GPU we used (NVIDIA RTX 3080). On the other hand, the simulation carried out using the GBION model took a few days using the same computational resources. Also note that the unexpected stacked state would have been missed in a typical explicit solvation simulation designed to explore the expected condensation pattern; the solvent box size (black dashed outline in Figure 8) would have been just large enough to accommodate the expected lateral condensed state, too small for the stacked one.

## 4 Methods

### 4.1 GBION Implementation Notes

**Figure 9:**
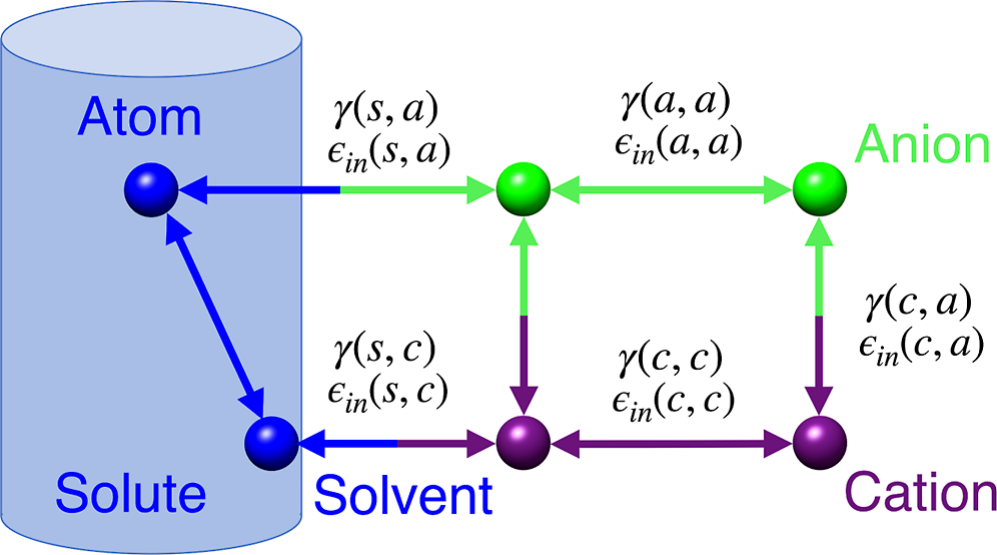
Detailed overview of the general implementation of the GBION framework for MD simulations. The specified coefficients are incorporated into eq 6.

The proposed framework can be effectively implemented in any computational environment that supports the general Born model. A key feature of this implementation is the differentiated treatment of various interacting atomic pairs. Specifically, each interacting atomic pair is characterized by unique coefficients, denoted as γ(a,b) and ϵ_in_(a,b), as shown in Figure 9.

This implementation involves a modified equation for calculating the electrostatic interactions between atomic pairs
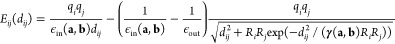
6

The current implementation details within the AMBER software package are provided in Supporting Information. Our analysis indicates that, in general, GBION parameters in the following range yield an accurate representation of the modeled ion distributions:

- γ(s,a), γ(a,a), and γ(c,c) are approximately 4, maintaining the canonical GB approximation for solute–anion, anion–anion, and cation–cation interactions.
- γ(s, c) and γ(a, c) are near 0, aligning solute–cation and cation–anion electrostatic interactions closely with Coulomb’s law.
- ϵ_in_(s, a) and ϵ_in_(a, a) are slightly lower than, but close to, the dielectric constant of water (ϵ_*w*_).
- ϵ_in_(s, c), ϵ_in_(a, c), and ϵ_in_(c, c) for monovalent ions are approximately equal to .

The specific values of these coefficients depend on other parameters within the employed GB model as well as on the particular DNA and ion models used in the implementation and on the ion charge.

#### 4.1.1 Optimization of the Model Parameters

The following parameters were optimized for best fit against the reference explicit solvent values and distributions: radial distributions of sodium and potassium ions and the degree of the DNA charge neutralization by CoHex^3+^ ions. We carried out a number of simulations with different values of GBION parameters to find the sets of parameters that best reproduce the explicit solvent distributions of ions around the DNA. Each trajectory was made with the protocol described below. During the process of searching through the parameter space, we have revealed the following trends, which helped with the optimization process:

- The larger the *K*_GB_(s, *) (see Supporting Information for implementation details within AMBER), the stronger the ions bind to the phosphate backbone of DNA. Variation of *K*_GB_(s, *) modulates the height of the second peak of ion distribution around DNA (see Figure 5)
- The larger the *K*_ϵ_(s, *) is, the better the ions accumulate in the ion-binding sites of the DNA structure
- The larger the *K*_ϵ_(c, c) is, the smaller the absolute value of affinity of cations to DNA is–the stronger the ions repel from each other, so if some ions are already bound to the DNA, additional ions tend to bind less frequently.

Using the aforementioned trends as guidance, we varied the parameters *K*_GB_ in the range of 0–2 and *K*_ϵ_ in the range of 1–78.5 to find the optimal values reported below in the simulation protocols.

### 4.2 Simulation Protocols

#### 4.2.1 DNA–NaCl and DNA–KCl Systems: Explicit Water MD Simulations

The explicit water MD simulations of the 25 bp-long DNA duplex with the sequence (GCATCTGGGCTATAAAAGGGCGTCG) in solutions of NaCl and KCl were carried out using the bsc1 DNA force field using AMBER 18. The 25 bp duplex is constructed in the canonical B-form using Nucleic Acid Builder.^144^ The DNA duplex was solvated with 29255 OPC water molecules, 104 cations (Na^+^ or K^+^), and 56 Cl^–^ ions. For the simulation of DNA with the NaCl and SPC/E water model 29213 water molecules, 103 Na^+^ ions and 55 Cl^–^ ions were used. The truncated octahedron was set as a form of simulation box to approximate the results obtained using spherical restraints. To calculate how many ions we should add to the system to reach the desired bulk salt concentration (0.145 M), we used the SLTCAP^132^ method^d^. We used the OPC water model^137^ and Joung/Cheatham (J/C) ion parameter set^138^ for reference simulations. This solvent model was found earlier to be the best compromise choice for the reproduction of the experimental Na^+^ and K^+^ distributions around DNA.^136^ The same ion parameter set^138^ was used in SPC/E simulation. First, we ran 2000 step-long initial minimization using the steepest descent algorithm for the first 1000 steps and the conjugate gradient algorithm for 1001–2000 steps. After the initial minimization, each system was heated from 0 to 300 K in a canonical ensemble (*NVT*) for 18 ps and then equilibrated for 2 ps in the same ensemble. Then, it was equilibrated for 40.06 ns in isothermal–isobaric ensemble (*NPT*) using Langevin dynamics with a collision frequency of 2 ps^–1^ to reach 1 atm pressure. Periodic boundary conditions and the particle mesh Ewald method were used. After the equilibration, 300 ns long production trajectories were generated for each system using the *NPT* ensemble. The integration time-step was 2 fs. During the heating and equilibration, every atom of 25 bp DNA was restrained to their original positions with harmonic restraints with a 5 kcal/mol/Å^2^ force constant. During the production simulation, the constant was 0.01 kcal/mol/Å^2^. Snapshots were saved every 10 ps along each trajectory. We use these simulations to examine the ion distributions around DNA alone, so the DNA was restrained to avoid the influence of DNA conformation change on ion distribution.

#### 4.2.2 DNA–NaCl and DNA–KCl Systems: Implicit Water/Explicit Ions GBION Simulations

A 25 bp DNA duplex with the sequence (GCATCTGGGCTATAAAAGGGCGTCG) was constructed as described above for the explicit water simulations. The DNA was simulated with NaCl and KCl using the OL15 DNA force field. Ion parameters for Na^+^, K^+^, and Cl^–^ are taken from the Joung/Cheatham (J/C) set for the OPC water model,^138^ and mbondi3 radii set was used; all the parameters of the ions are presented in Table S1. To match the concentrations of NaCl and KCl achieved in the explicit solvent simulations, the same amounts of ions (104 cations and 56 anions) were added to the systems simulated using the GBION model. For the enlarged restraining sphere, with a radius of 120 Å, 458 cations and 410 anions were added. The parameters of the GBION model for the DNA–NaCl simulations are listed below [K_*_(a,b) = parameter name as implemented in AMBER = its value, see Supporting Information for implementation details within AMBER]. In what follows, s = solute atoms, c = cation, and a = anion.

1. *K*_GB_(s, c) = gi_coef_1_p = 1
2. *K*_GB_(s, a) = gi_coef_1_n = 0.05
3. *K*_GB_(c, c) = gi_coef_1_pp = 1
4. *K*_GB_(a, c) = gi_coef_1_pn = 0.05
5. *K*_GB_(a, a) = gi_coef_1_nn = 1
6. *K*_ϵ_(s, c) = intdiel_ion_1_p = 54
7. *K*_ϵ_(s, a) = intdiel_ion_1_n = 8
8. *K*_ϵ_(c, c) = intdiel_ion_1_pp = 54
9. *K*_ϵ_(a, c) = intdiel_ion_1_pn = 8
10. *K*_ϵ_(a, a) = intdiel_ion_1_nn = 8

For simulations with KCl, the parameters are the same except *K*_ϵ_(s, c) = 36 and *K*_ϵ_(c, c) = 36. First, we ran 2000 step-long initial minimization using the steepest descent algorithm for the first 1000 steps and the conjugate gradient algorithm for 1000–2000 steps. After the initial minimization, all of the systems were heated from 0 to 300 K over 20 ps. Each system was equilibrated for 0.4 ns using Langevin dynamics with a collision frequency of 0.05 ps^−1^. After the equilibration, 10 ns long production trajectories were generated for each system. The integration time-step was 2 fs. Unless otherwise specified, the nonpolar surface energy term (GB/SA) was not accounted for in the simulations (gbsa = 0). When GB/SA calculations are applied (gbsa = 3), the nonpolar term in eq 1 is proportional to the SASA of the solute, while the GB model is used to calculate the electrostatic term in eq 1. Distributions do not depend on taking into account the surface energy, see Figure S6. During the heating, equilibration, and production simulation, every atom of 25 bp DNA was restrained to their original positions with a 0.01 kcal/mol/Å^2^ force. Snapshots were saved every 1 ps along the production trajectory. To limit the simulation volume, we applied the restraining potential that works as follows. If an ion’s position exceeds a specified distance from the attractive center (in this case 67.7 Å from the 401th atom of the DNA duplex, which is atom C4 of the 13th residue, thymine), a harmonic restraining potential with a constant of 20 kcal/mol/Å^2^ begins to act on the ion. For ions closer to the center than that distance, the restraining force is zero. A confining sphere with a radius of 67.7 Å was chosen to match the NaCl and KCl concentrations in the equilibrated truncated octahedron used in the explicit water simulations described above. The robustness of the ion distribution with respect to variations of the restraining sphere’s radius was verified, see Figure S7.

The dielectric constants of the solutes and the solvent were chosen as ϵ_in_ = 1 and ϵ_out_ = 78.5, respectively. The DNA duplex atomic partial charges and the parameters for van der Waals interactions between the DNA, Na^+^, and Cl^–^ ions are taken from the AMBER *OL15* force field.^145^

##### 4.2.2.1 Dickerson–Drew Dodecamer Simulations

To verify that the DNA remains stable during GBION simulations, we simulated Dickerson–Drew dodecamer (CGCGAATTCGCG) for 1 μs. To limit the simulation volume, we applied the restraining potential that works as follows. If an ion exceeds a specified distance of 40 Å from the center of mass of phosphorus atoms of the DNA backbone, harmonic restraining potential with a constant of 20 kcal/mol/Å^2^ begins to act on the ion. For ions closer to the center than that distance, the restraining force is zero. In this case, the center of mass, as opposed to a single atom, was chosen as the reference center point to avoid a significant influence of the restraints on the DNA structure. The number of cations in this case is 36, anions −14. The parameters of minimization, heating, and equilibration simulations were the same as for 25 bp-long DNA duplexes. Production simulation parameters were the same except for the DNA restraints and simulation time. During the 1 μs production simulation, the DNA atoms were not restrained. Snapshots were saved every 100 ps along the production trajectory. The integration time-step was 2 fs.

#### 4.2.3 DNA–CoHex^3+^ System: Explicit Water MD Simulations

The details of the all-atom MD simulations of DNA–CoHex^3+^ systems using the explicit TIP3P water model^146^ are described elsewhere^31^ and are similar to the all-atom MD simulation of the DNA–NaCl and DNA–KCl systems described above.

#### 4.2.4 DNA–CoHex^3+^ System: Implicit Water/Explicit Ions GBION Simulations

The construction of the 25 bp homopolymeric (GCATCTGGGCTATAAAAGGGCGTCG) DNA duplex is described above. The duplex (charge −48*e*) was neutralized by 16 CoHex^3+^ ions. Ion parameters are taken from ref (31). No other ions were included in the simulations. The DNA force field was the same as in the case of the DNA–NaCl and DNA–KCl systems described above (OL15). The parameters of the GBION model for DNA–CoHex^3+^ simulations are listed below [K_*_(a,b) = parameter as implemented to AMBER = value]:

1. *K*_GB_(s, c) = gi_coef_1_p = 0.01
2. *K*_GB_(s, a) = gi_coef_1_n = 0.01
3. *K*_GB_(c, c) = gi_coef_1_pp = 0.06
4. *K*_GB_(a, c) = gi_coef_1_pn = 0.06
5. *K*_GB_(a, a) = gi_coef_1_nn = 0.06
6. *K*_ϵ_(s, c) = intdiel_ion_1_p = 1
7. *K*_ϵ_(s, a) = intdiel_ion_1_n = 1
8. *K*_ϵ_(c, c) = intdiel_ion_1_pp = 2
9. *K*_ϵ_(a, c) = intdiel_ion_1_pn = 2
10. *K*_ϵ_(a, a) = intdiel_ion_1_nn = 2

The equilibration step in the simulations of DNA with CoHex^3+^ ions was 500 ps long. During the equilibration, every atom of the DNA was restrained to its initial position with a 1 kcal/mol/Å^2^ harmonic restraining potential. The production simulation was 10 ns long. During the production run, the DNA atoms were also restrained with 0.01 kcal/mol/Å^2^ to match the conditions of the explicit solvent simulation. The integration time-step was 2 fs.

##### 4.2.4.1 Simulation of Ion-Induced DNA Condensation

To simulate counterion-induced aggregation of a pair of DNA duplexes, we used the same structure as that for the single DNA simulations. Two copies of the system simulated above were initially located at a distance of 74 Å from each other. The minimization and heating simulations were the same as those above. The equilibration step was 50 ps long, and the simulation time step for this equilibration step only was chosen as 0.5 fs to avoid possible numerical instability. The integration time-step was 2 fs in all of the following simulation steps. Production simulation was 300 ns long. During the production simulation, the DNA duplexes were not restrained. To limit the simulation volume, we applied the restraining potential to the ion if the distance from the 413rd atom of one of the duplexes exceeded 160 Å (in other words, the ion-restraining sphere was centered on one of the duplexes).

### 4.3 Trajectory Analysis and Calculation of Ion Distributions

For analysis of ion distributions around DNA, we have used Curves+ and Canion^147^ programs. Curves + translate coordinates into a convenient curvilinear helicoidal system. Canion analyzes the curvilinear coordinates of ions and generates 1D and 2D distributions and density maps of ions around DNA. To calculate groove widths, rmsd of DNA, and WC values, we used the program *nastruct*, included in the AMBER package.

### 4.4 Calculating the Number of Ions Associated with the DNA

As an experimental reference, here we utilized the findings of Bai et al., who measured the number of ions associated with DNA using the buffer exchange technique.^134^ Their experiments showed that the number of ions associated with double-stranded DNA remains relatively constant across bulk salt concentrations ranging from 0.1 to 0.2 M. To align with our study, the experimental value for the number of DNA-associated ions was scaled by a factor of 24/23, reflecting the ratio of the total charge between our 25 base-pair DNA fragment and the 24 base-pair fragment used in the experiment. For a direct comparison with the experimental results, we calculated the number of DNA-associated ions as follows^148^
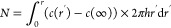
7where *c*(∞) represents the bulk salt concentration, determined as the mean concentration between 28 and 30 Å from the DNA helical axis in this work, and *h* indicates the length of the DNA duplex, which is 85 Å in our case. The ion concentration as a function of distance from the DNA helical axis, *c*(*r*), was computed using the Curves+ and Canion programs.^147^

### 4.5 Generating Schematic Graphs of Electrostatic Energy between Charged Atoms

To illustrate the dependence of function *E*_*ij*_(*d*_*ij*_) on its parameters ϵ_in_(a,b) and γ(a,b), Figure 3b, for a generic pair of positive and negative charges of value ±1, we have used the following generic function
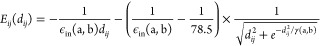
8

For Coulomb’s law, we used the generic function
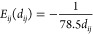
9

In eq 8, the Born self-terms were assumed constant (set to zero for simplicity), and the effective Born radii were set to 1. This, by no means general, approximation reflects the fact that the effective Born radii for the pair of small ions change relatively little over the relatively small range of *d*_*ij*_ of interest to us here. Also, our focus is on the general behavior of *E*_*ij*_, eq 3, which is not affected by the approximation. The specific coefficients used as examples in that figure were chosen to generate distinguishable graphs.

### 4.6 Software Used

We used the AMBER^145^ program package for MD simulations. The GBION model used here is available in AMBER 24, released in April of 2024. For visualization of structures and trajectories, we used PyMol^149^ and CHIMERA.^150^ We used Curves+, Canion,^147^ and Python3 libraries NumPy^151^ and Matplotlib^152^ for ion distribution analysis and graph visualization, respectively. We used ChatGPT 4^153^ for style editing.

## 5 Conclusions

The ionic atmosphere around a biomolecule can have a significant effect on its structure, dynamics, and function, necessitating accurate yet efficient representation of ions in simulation. Existing solvent models face various limitations with respect to representing ions in atomistic simulations: explicit solvent models can be computationally expensive, while the “engine” of the more efficient implicit solvent MD simulations, the GB model, provides only a crude, mean field approximation for the ionic atmosphere.

In this study, we have made substantial modifications to the framework of the canonical GB approximation to explicitly take into account ions around the solute molecule. The key insight is to consider distinct functional forms for the solute–solute, solute–ion, and ion–ion interactions.

The resulting “Implicit Solvent with EXplicit Ions” framework was implemented in AMBER (version 24, released on 04/30/2024, or later version) as a GBION model and evaluated through extensive MD simulations of DNA duplexes surrounded by either monovalent (Na^+^, K^+^, and Cl^–^) or trivalent (cobalt hexammine, CoHex^3+^) ions. The computed ion distributions obtained from GBION simulations showed reasonable agreement with those estimated by traditional all-atom MD simulations in explicit water as well as in experiment, where available. The largest deviation between GBION and the explicit solvent distribution was about ∼ *k*_B_*T* (in energy units), comparable to relevant differences between ion distributions obtained using different established explicit water models.

The GBION model accurately captures key features of CoHex^3+^ distribution around double-stranded DNA, including the preferential ion binding to the “outer surface” of the B-DNA backbone and the ∼90% degree of DNA charge neutralization. In contrast to the canonical GB, the new approach can simulate the process of DNA condensation induced by multivalent counterions. Not only do these simulations yield the well-known “lateral” DNA condensation mode, but they also reveal an unexpected “stacking” condensation of DNA duplexes, supported by recent experimental evidence.^33^ The efficiency of GBION in simulating large solvent volumes without severely compromising computation efficiency enables the observation of the transition from the lateral to the “stacking” condensed mode, which would have been highly challenging to simulate in the traditional explicit solvent.

Below is a brief summary of the key advantages and limitations of GBION.

### 5.1 Advantages vs Canonical GB

In contrast to the canonical GB, the GBION model allows simulating ions explicitly; this level of detail is desirable for many, if not most, biologically relevant systems on time scales that are becoming routine today. In particular, the canonical GB does not distinguish between Na^+^ and K^+^, but GBION does. The model can also handle multivalent ions naturally, which is completely out of reach to the canonical GB.

### 5.2 Advantages vs Traditional Fully Explicit Solvent

The GBION model inherits the key advantage of the canonical GB model relative to the explicit solvent: the speed of conformational sampling. Specifically, GBION simulations sample conformational space 1–2 orders of magnitude faster than the corresponding explicit solvent simulations.

Another notable advantage of the GBION model over the traditional explicit solvation is that the GBION computational cost is not very sensitive to the size of the simulation volume. This unique feature of the approach allows one to simulate physiologically relevant, very low salt concentrations of some ions without the exorbitant computational expense that such simulations would incur in the explicit solvent due to the need for a very large solvent box.

### 5.3 Limitations

With the main focus of this work being conceptual development of the new model, the set of molecular systems used for the current parameterization and testing is limited. Specifically, we optimized parameters for Na^+^, K^+^, Cl^–^, and CoHex^3+^ to work with the DNA. The current parameters of the model will likely work reasonably well for other negatively charged solutes, but we have not verified that yet. We have not yet tested the ability of GBION to reproduce ion distributions around proteins; positively charged solutes may be particularly challenging to the current parameterization of Cl^–^. Therefore, we advise against using the current set of GBION parameters for protein simulations, especially those that carry a net positive charge. Also, biologically relevant divalent ions such as Mg^2+^ or Ca^2+^ have not yet been parameterized.

## Supporting Information Available

Comparison of sodium distributions obtained using the SPC/E water model with those presented in the text; capped radial-angle distributions of Na^+^ and K^+^; comparison of Cl^–^ distributions and dependence of cation distributions on the simulation times, taking into account solvent accessible surface energy and the radius of the restraining sphere; details of the model implementation in the AMBER package; and parameters of monovalent ions used in the simulations (PDF)
Movie of 225 ps-long Dickerson–Drew dodecamer simulation with KCl (MP4)
Movie of 100 ns-long simulation of DNA condensation in the presence of CoHex^3+^ (MP4)

## Footnotes

a Proof-of-concept MD simulations based on the numerical PB were reported,^43,80−82^ including an explicit ion model.^83^ It is believed that the inherent algorithmic complexity of the numerical PB approach, conceptual difficulties associated with incorporating PB-based algorithms into MD,^84^ and significant computational expense^85^ preclude its wider adoption in atomistic MD.

b Physically, the interaction *E*_*ij*_ between solvent-separated charges must be zero in the conductor limit of ϵ_out_ → *∞*, but this is not the case for the canonical GB. In contrast, the pure Coulomb interaction has the correct limiting behavior.

c Every dimension of the explicit solvent box needs to be at least 180 Å, which translates into ∼2 × 10^5^ water molecules or ∼7 × 10^5^ atoms. According to recent AMBER benchmarks, the simulation speed of a system containing ∼4 × 10^5^ atoms is ∼45 ns/day (NVIDIA RTX 3080, NPT ensemble, 2 fs step). Extrapolating to ∼7 × 10^5^ atoms yields ∼25 ns/day. Consequently, a 4 μs-long simulation of the system containing ∼7 × 10^5^ atoms should take at least ∼0.5 years.

d The actual bulk salt concentration inferred from an analysis of the ion distributions (Figure 5) is ∼0.1 M, which is slightly less than 0.145 M targeted by SLTCAP. However, since the difference with the target is small and 0.1 M can still be considered physiological, we did not apply further corrections.
