# Supplementary material for: Implicit Solvent with Explicit Ions Generalized Born Model in Molecular Dynamics: Application to DNA
Source: J Chem Theory Comput. 2024 Sep 16;20(19):8724–39. doi: 10.1021/acs.jctc.4c00833 (PMC11465471; doi:10.1021/acs.jctc.4c00833)
Supplement: Supplementary file 1 — ct4c00833_si_001.pdf [file ct4c00833_si_001.pdf]

Supplementary information for: Implicit Solvent with Explicit Ions Generalized Born model in Molecular Dynamics: Application to DNA

Egor S. Kolesnikov,[†,‡] Yeyue Xiong,[¶,‡] and Alexey V. Onufriev[*,§]

†*Department of Physics, Virginia Tech, Blacksburg, VA 24061, USA*

‡*Authors made equal contribution.*

¶*Department of Biomedical Engineering and Mechanics, Virginia Tech, Blacksburg, VA 24061, USA*

§*Departments of Computer Science and Physics, Center for Soft Matter and Biological Physics, Virginia Tech, Blacksburg, VA 24061, USA*

E-mail: alexey@cs.vt.edu

Supporting Text

Supporting Figures S1-S7

Supporting Table S1

Na^+ distributions around DNA in GBION vs. two explicit water models

In Figure S1 we compare the magnitude of deviations of the GBION ion distributions from the explicit solvent reference (OPC) with the corresponding deviations between two explicit solvent models: OPC and SPC/E. These two explicit water models were previously found to produce noticeably different distributions of monovalent ions around dsDNA.[1]

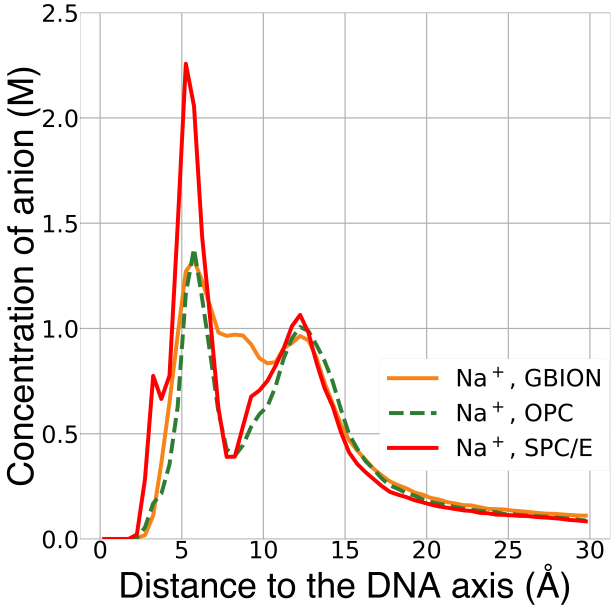

Figure S1: The deviations between GBION and OPC - based distribution of Na^+ around DNA can be as high as the deviations between the corresponding distributions produced by two established explicit water models, OPC and SPC/E.

(a) Na^+, `GBION` model

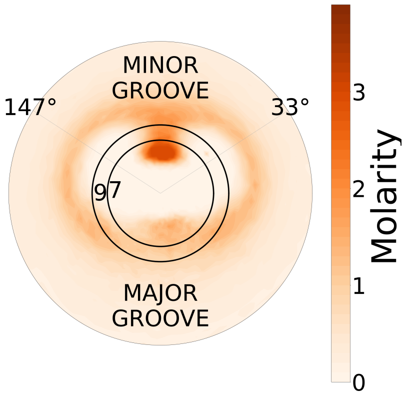

(b) Na^+, explicit solvent

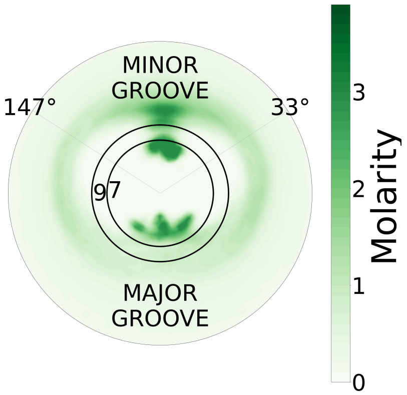

(c) K^+, `GBION` model

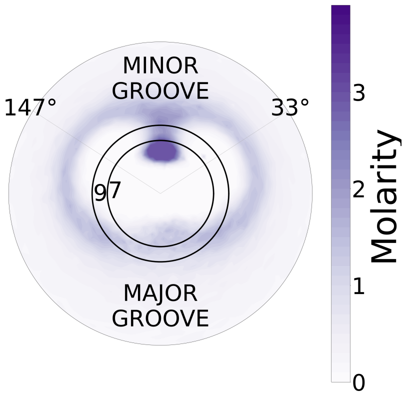

(d) K^+, explicit solvent

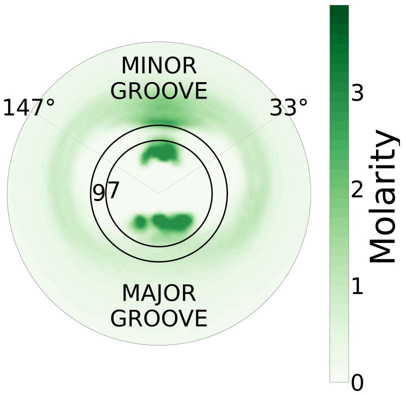

Figure S2: Radial-angle distributions of Na^+ and K^+ around DNA capped at 3M: for emphasis, all the values exceeding 3M were set to 3M. The water model used to obtain each distribution is indicated above each panel.

The distribution of Cl^- around DNA

No specific fitting of Cl^- parameters in `GBION` was performed. The existing parameters of Cl^- yield the expected distribution of the ions around the negatively charged DNA, but we stress that no testing of these Cl^- parameters in the context of a positively charged solute was performed.

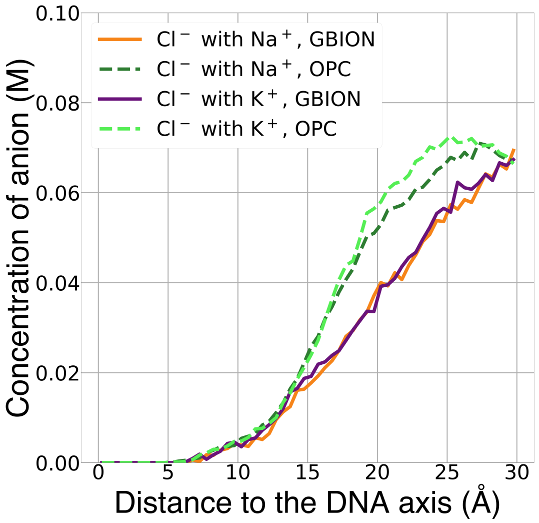

Figure S3: The distributions of Cl^- around DNA in the presence of Na^+ and K^+, obtained using `GBION` (orange for NaCl solution and purple for KCl) model and the explicit OPC water model (dashed dark green for NaCl solution and dashed light green for KCl) are close to each other. Cl^- ions repel from the DNA, as they should, due to like charge electrostatic repulsion.

Convergence of `GBION` distributions

To estimate the sensitivity (convergence) of `GBION` -derived ion distributions to simulation time, we compared cation distributions around dsDNA averaged over various time intervals: 10 ns, 1 ns, 100 ps, and 10 ps. To this end, we have simulated the DNA in the presence of NaCl and KCl using the `GBION` model, for 10 ns and 100 ps, with 10000 frames saved from each simulation. As a result, we generated four distinct trajectories: 100 ps with KCl, 100 ps with NaCl, 10 ns with KCl, and 10 ns with NaCl. We then compared the distributions of Na^+ and K^+ ions averaged over the following:

- The entire 10 ns simulations of DNA with NaCl and KCl (10000 frames)
- The first 1 ns of the 10 ns simulations of DNA with NaCl and KCl (1000 frames)
- The entire 100 ps simulations of DNA with NaCl and KCl (10000 frames)
- The first 10 ps of the 100 ps simulations of DNA with NaCl and KCl (1000 frames)

See Figure S4. For the explicit solvent model (OPC), we compare the distributions averaged over the first 100 ns of the simulations with those averaged over the whole 300 ns of the simulation.

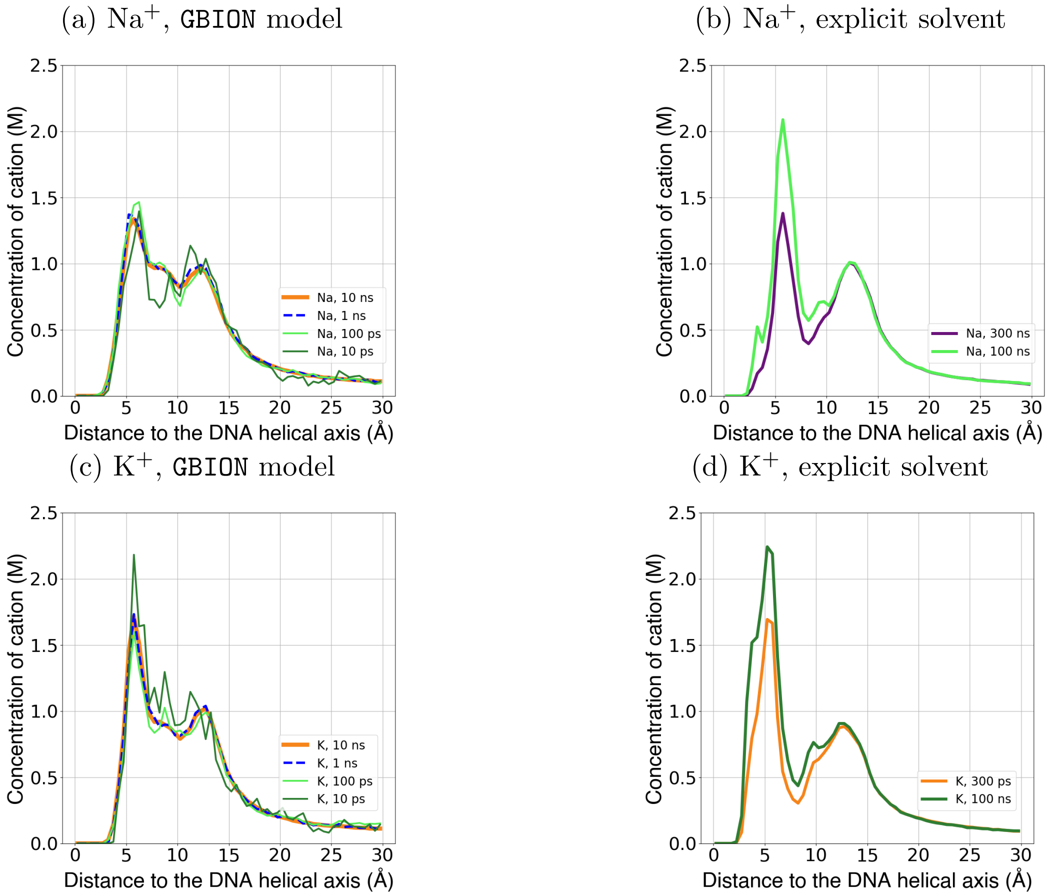

Figure S4: Na^+ and K^+ distributions around DNA obtained using GBION model are well converged at 1 ns. Shown are the distributions of Na^+, obtained using GBION model (a) and using explicit water OPC (b), averaged over different simulation times. Distributions of K^+ obtained using GBION model (c) and using explicit water OPC (d) are averaged over different simulation times, as indicated in the insets.

Implementation in AMBER

The current implementation of the Implicit Solvent with EXplcit Ions framework, `GBION` , in AMBER package is optimized to be used in combination with GBneck2 GB model.[2] In GBneck2 model, a correction is used to eliminate interstitial regions of high dielectric smaller than the solvent molecule. The contribution of this neck correction is defined by the parameter *gbneckscale*. We have implemented `GBION` , Eq. 3 (main text), in the form of Eq. 1. This implementation allows for different atom pair types to have different $\gamma(a, b)$ and $\epsilon_{in}(a, b)$, which translate into the scaling coefficients $K_{GB} = \gamma(a, b)/4$ and K_ϵ (we have also implemented an option to scale the parameter *gbneckscale* by coefficient K_{NS}, but it is unused in this optimization, *i.e.* $K_{NS} = 1$). See the list of the implemented and used coefficients in Fig. S5. For K_{GB}, one can set different values for different pairs of interacting atoms: $K_{GB}(solvent - cation)$, $K_{GB}(solvent - anion)$, $K_{GB}(cation - cation)$, $K_{GB}(cation - anion)$, and $K_{GB}(anion - anion)$. The same applies to K_ϵ. K_{GB} and K_ϵ are coefficients that modulate scale factor for the product of Born radii and dielectric constant respectively. The default values of all Ks are 1: *i.e.* if all the $K = 1$, `GBION` is reduced to the canonical GB. Note that the solute-solute parameters remain unchanged: these are determined by the underlying GB model used. The idea is that this part of the interaction potential has already been optimized. Other parameters are optimized specifically in the context of `GBION` model.

$$\Delta G_{\text{el}} = -\frac{1}{2} \sum_{i,j,\mathbf{a},\mathbf{b}} \left(\frac{1}{\mathbf{K}_\epsilon(\mathbf{a}, \mathbf{b})\epsilon_{in}} - \frac{1}{\epsilon_{out}} \right) \frac{q_i q_j}{\sqrt{d_{ij}^2 + R_i R_j \exp\left(-d_{ij}^2/(4R_i R_j \mathbf{K_{GB}}(\mathbf{a}, \mathbf{b}))\right)}} \quad (1)$$

Here, index $(\mathbf{a}, \mathbf{b})$ denotes the type (topology) of the pair of interacting atoms (solute-cation, solute-anion, cation-anion and so on), see Fig. S5. The way the effective Born radii R_i are estimated, which defines the "flavor" of the GB model, are computed here via the most recent GB implementation in AMBER, "gb-neck2" (igb=8 in AMBER input file).[3–5]

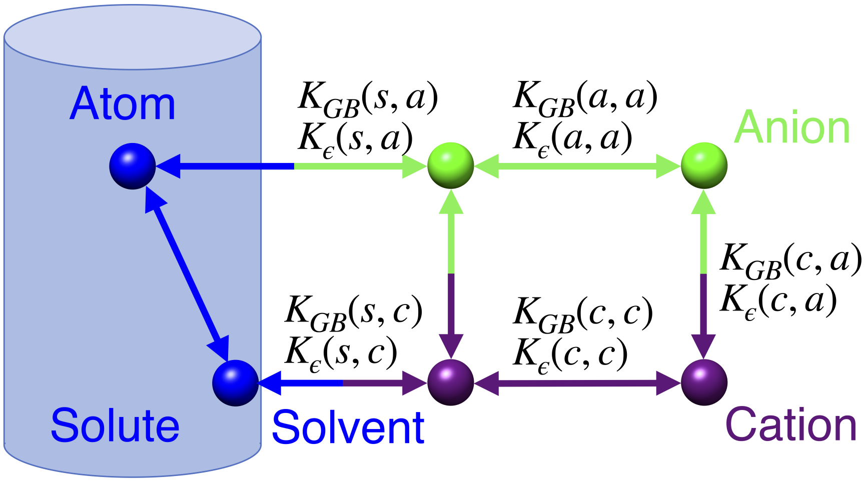

Figure S5: The details of the implementation of `GBION` framework for MD simulations in AMBER, GPU and CPU versions. The coefficients listed are implemented in Eq. 1.

The atomic radii set appropriate for gb-neck2 is used, that is mbondi3. In particular, the radius of Na and K is 1.5 Å , and those of the atoms of CoHex are: Co 1.7 Å, N 1.55 Å and H 1.5 Å.

We note that the formulation of our framework is agnostic to the "base" GB model used, any GB flavor can be paired with it just as well (with its own set of `GBION` parameters). The present `GBION` model parameterized to work with igb=8 is available in AMBER package, version 24 , released on 04/30/2024, or a later version.

Ion parameters used in the simulations

Table S1: Parameters of ions used in all the GBION simulations.

	Ion type		
Parameter	Na^+	K^+	Cl^-
σ	1.307	1.516	2.103
ϵ	0.0296	0.1395	0.6788
Mass	22.99	39.1	35.45
Charge	+1	+1	-1
Atomic Radius	1.5	1.5	1.7
GB Screen	0.8	0.8	0.8

The effect of the surface energy term

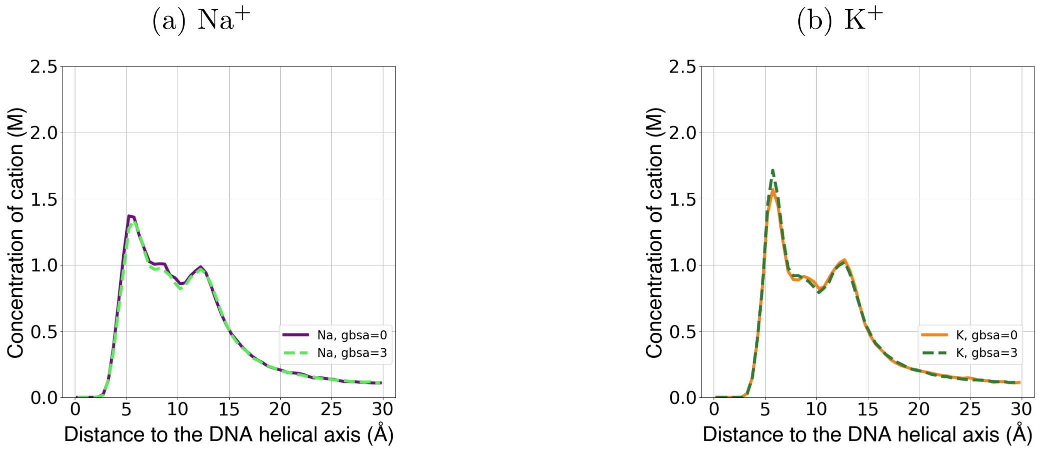

Figure S6: (a) Distributions of Na^+ around DNA obtained using GBION model with the surface energy term present (gbsa=3) and without (gbsa=0). (b) Distributions of K^+ obtained using GBION model with the surface energy term present (gbsa=3) and without (gbsa=0).

The effect of the size of the restraining sphere

(a) Na^+ (b) K^+

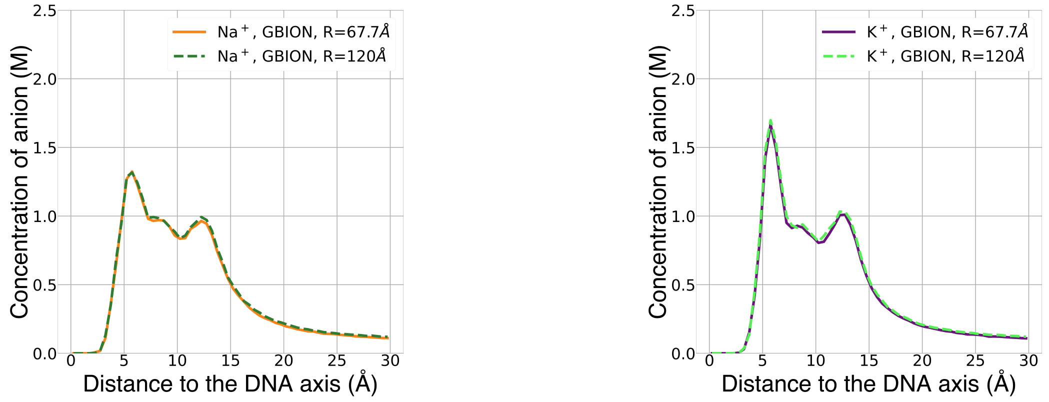

Figure S7: Distributions of Na^+ around DNA obtained using the `GBION` model with different radii of the restraining sphere. (b) Distributions of K^+ obtained using the `GBION` model with different radii of the restraining sphere. The distributions remain stable when the radius of the restraining sphere is varied.

References

(1) Kolesnikov, E. S.; Gushchin, I. Y.; Zhilyaev, P. A.; Onufriev, A. V. Similarities and Differences between Na+ and K+ Distributions around DNA Obtained with Three Popular Water Models. *J. Chem. Theory Comput.* **2021**, *17*, 7246–7259.

(2) Mongan, J.; Simmerling, C.; McCammon, J. A.; Case, D. A.; Onufriev, A. Generalized Born Model with a Simple, Robust Molecular Volume Correction. *Journal of Chemical Theory and Computation* **2007**, *3*, 156–169.

(3) Nguyen, H.; Roe, D. R.; Simmerling, C. Improved Generalized Born Solvent Model Parameters for Protein Simulations. *J. Chem. Theory Comput.* **2013**, *9*, 2020–2034.

(4) Nguyen, H.; Pérez, A.; Bermeo, S.; Simmerling, C. Refinement of Generalized Born Implicit Solvation Parameters for Nucleic Acids and Their Complexes with Proteins. *J. Chem. Theory Comput.* **2015**, *11*, 3714–3728.

(5) Tian, C.; Kasavajhala, K.; Belfon, K. A. A.; Raguette, L.; Huang, H.; Migues, A. N.; Bickel, J.; Wang, Y.; Pincay, J.; Wu, Q.; Simmerling, C. ff19SB: Amino-Acid-Specific Protein Backbone Parameters Trained against Quantum Mechanics Energy Surfaces in Solution. *J. Chem. Theory Comput.* **2020**, *16*, 528–552.
